# Supplementary material for: Relative income within the household, gender norms, and well-being
Source: PLoS One. 2024 Oct 25;19(10):e0306180. doi: 10.1371/journal.pone.0306180 (PMC11510123; doi:10.1371/journal.pone.0306180)
Supplement: S1 Appendix — (PDF) [file pone.0306180.s001.pdf]

Appendix A: Supplemental Figures and Tables

Figure A.1: Gender Norms, Well-being and Health, Men

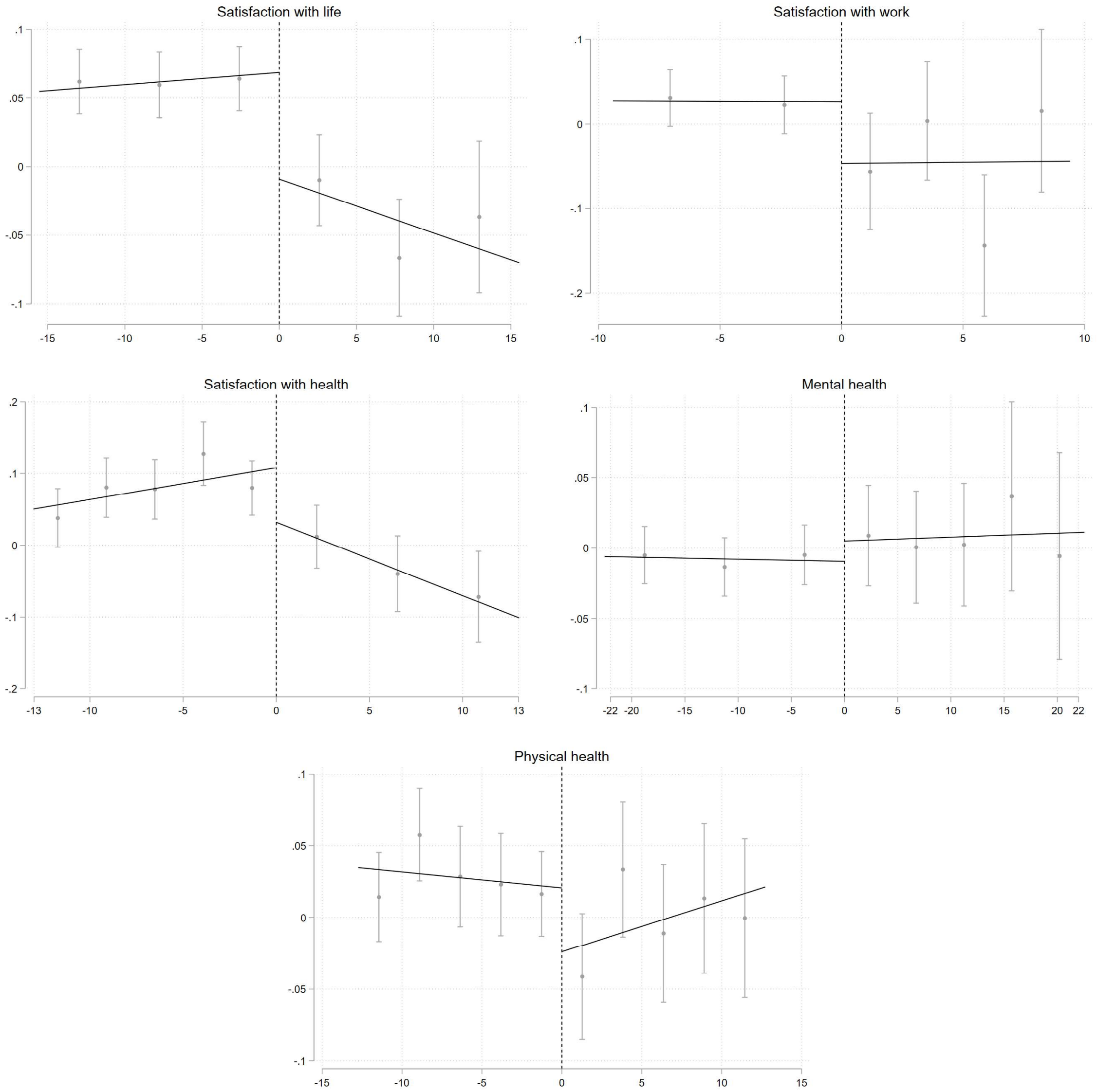

Notes - This figure describes the relationships between satisfaction in different life domains, health (mental and physical) among men and the gender income differences within households. The standard errors are clustered by relative income bins (in €1,000 intervals). All specifications include individual fixed effects. For bandwidth and binning selection, we use the MSE-optimal bandwidth and the IMSE-optimal evenly-spaced method (Calonico et al., 2022). 95% confidence intervals are reported in bars.

Figure A.2: Gender Norms, Well-being and Health, Women

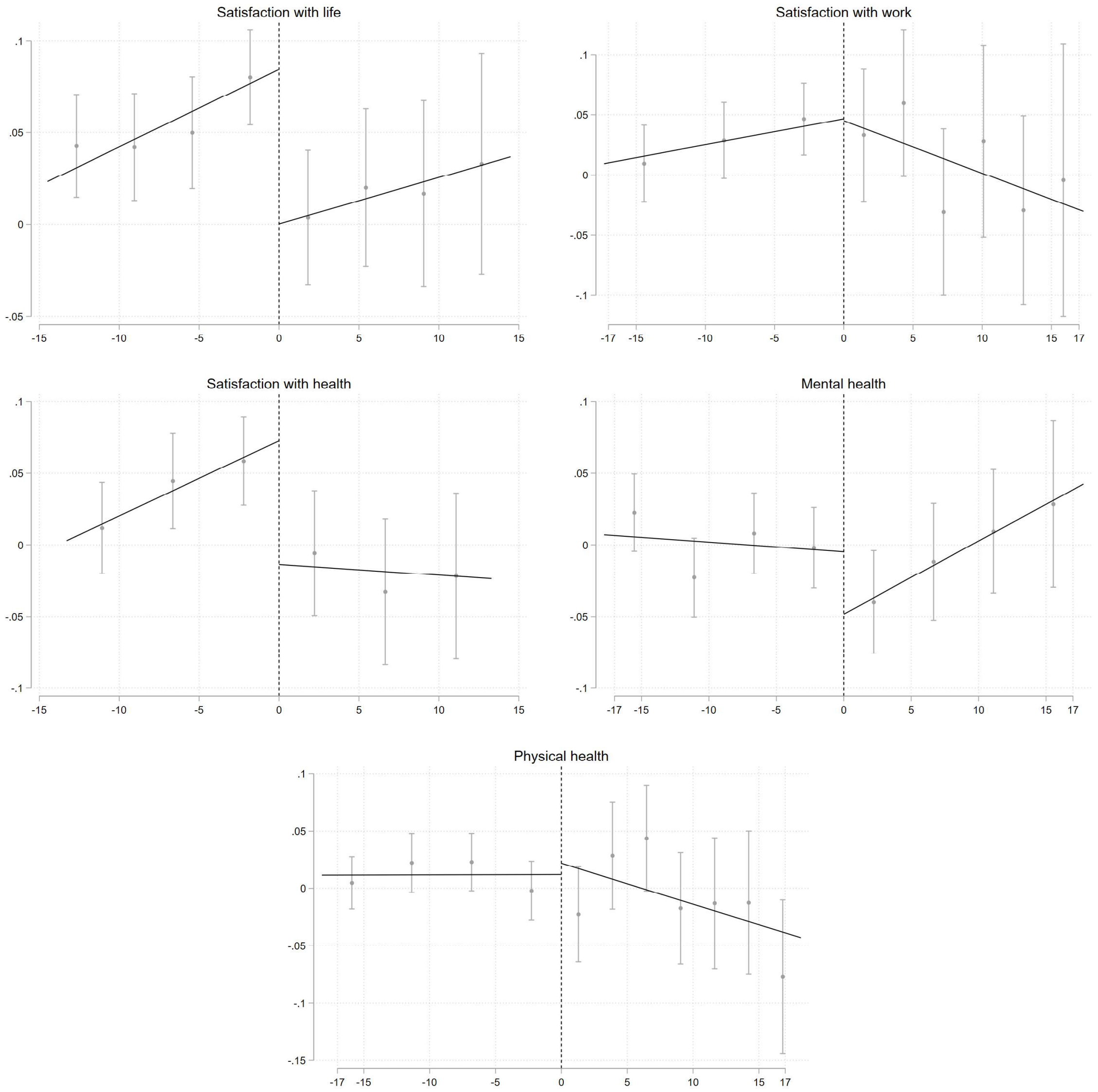

Notes - This figure describes the relationships between satisfaction in different life domains, health (mental and physical) among women and the gender income differences within households. All specifications include individual fixed effects. The standard errors are clustered by relative income bins (in €1,000 intervals). For bandwidth and binning selection, we use the MSE-optimal bandwidth and the IMSE-optimal evenly-spaced method (Calonico et al., 2022). 95% confidence intervals are reported in bars.

Table A.1: Descriptive Statistics

	Obs.	Mean	Std. dev.	Obs.	Mean	Std. dev.
	Men			Women		
Outcome variables						
Satisfaction with life	78,735	7.31	1.59	78,765	7.38	1.61
Satisfaction with family	40,224	8.29	1.53	40,267	8.20	1.61
Satisfaction with housework	48,831	7.00	1.90	68,785	6.73	1.85
Satisfaction with work	74,540	7.07	1.99	70,400	7.12	1.98
Satisfaction with sleep	35,891	6.95	2.05	35,911	6.76	2.15
Satisfaction with childcare	20,074	7.12	2.24	19,424	7.22	2.36
Satisfaction with health	78,585	6.91	2.00	78,608	6.94	2.02
Poor health	68,359	0.44	0.50	68,370	0.43	0.50
Mental health	24,465	51.65	8.60	24,412	50.04	9.22
Physical health	24,465	51.44	8.21	24,412	51.41	8.43
Control variables						
Wage	79,225	35,449	24,462	79,225	17,731	14,396
Age	79,225	45.21	9.40	79,225	42.51	9.31
College degree	79,225	0.35	0.48	79,225	0.29	0.45
Number of children	79,225	0.98	1.03			

Notes - Data are drawn from the SOEP (version 37) for individuals aged 18-64 years (survey years: 1984-2020). All the samples include married couples and contain individuals for whom information on all observables are not missing. The mental health and physical health variables are not standardized.

Table A.2: Balancing Tests – Control Variables

	(1) Males	(2) Females
**Panel A: Earnings difference in the first year of marriage**		
Age	-0.179	0.064
	(0.767)	(0.706)
College degree	-0.017	-0.022
	(0.029)	(0.017)
Observations	10,034	
**Panel B: Earnings difference over time**		
Age	-0.308	-0.057
	(0.312)	(0.294)
College degree	-0.012	-0.011
	(0.009)	(0.009)
Observations	79,225	

Notes - Standard errors are reported in parentheses and are clustered by relative income bins (in €1,000 intervals). In both Panels, we used covariates measured in the first year of marriage. In Panel A, the running variable is also measured in the first year of marriage. In Panel B, we instead use all the available years to construct the running variable. We used the optimal bandwidth using the mean square error (MSE) selection criteria and robust inference following Calonico et al. (2014).
*Significant at 10 per cent; ** Significant at 5 per cent; ***Significant at 1 per cent.

Table A.3: Balancing Tests – Outcome Variables

Dep. var.:	(1) Earnings difference in the first year of marriage	(2) Earnings difference over time
Satisfaction with life	-0.118	-0.182
	(0.368)	(0.114)
Satisfaction with family	-0.588	-0.068
	(0.830)	(0.243)
Satisfaction with housework	0.163	-0.068
	(0.457)	(0.117)
Satisfaction with work	-0.151	0.060
	(0.251)	(0.161)
Satisfaction with sleep	0.113	0.040
	(0.389)	(0.312)
Satisfaction with childcare	0.423	-0.165
	(0.488)	(0.216)
Satisfaction with health	0.138	-0.057
	(0.238)	(0.066)
Health status	0.142	-0.042
	(0.086)	(0.058)
Mental health	-3.525	-2.940
	(3.217)	(2.207)
Physical health	0.197	1.337
	(2.253)	(0.870)

Notes - Standard errors are reported in parentheses and are clustered by relative income bins (in €1,000 intervals). The outcomes measured in the first year available in the SOEP before marriage and restricted the sample to individuals under 45. In column 1, the running variable is measured in the first year of marriage. In column 2, we instead use all the available years to construct the running variable. We used the optimal bandwidth using the mean square error (MSE) selection criteria and robust inference following Calonico et al. (2014).
*Significant at 10 per cent; ** Significant at 5 per cent; ***Significant at 1 per cent.

Table A.4: RD Estimates: Gender Norms and Well-being - Including Individual FE

Satisfaction with	(1)<br>Life	(2)<br>Family	(3)<br>Housework	(4)<br>Work	(5)<br>Sleep	(6)<br>Childcare
			Panel A: Males			
RDD wife earns more	-0.074**	-0.059*	-0.029*	-0.066***	-0.064*	0.011
	(0.033)	(0.033)	(0.016)	(0.019)	(0.035)	(0.095)
Observations	78,735	40,224	48,831	74,540	35,891	20,074
Mean of dep. var.	7.187	8.279	6.868	7.108	6.902	7.075
Std. dev. of dep. var.	1.725	1.577	2.008	2.041	2.132	2.287
			Panel B: Females			
RDD wife earns more	-0.093***	-0.036	-0.050***	-0.013	-0.052	0.131**
	(0.017)	(0.029)	(0.016)	(0.033)	(0.049)	(0.062)
Observations	78,765	40,267	68,785	70,400	35,911	19,424
Mean of dep. var.	7.264	8.204	6.757	7.104	6.690	7.178
Std. dev. of dep. var.	1.732	1.655	1.906	2.061	2.214	2.408

Notes - Standard errors are reported in parentheses and are clustered by relative income bins (in €1,000 intervals). All specifications include individual fixed effects. For each outcome, we used the optimal bandwidth using the mean square error (MSE) selection criteria and robust inference following Calonico et al. (2014).
*Significant at 10 per cent; ** Significant at 5 per cent; ***Significant at 1 per cent.

Table A.5: RD Estimates: Gender Norms and Health - Including Individual FE

Dep. var.:	(1)<br>Satisfaction with health	(2)<br>Poor health	(3)<br>Mental health	(4)<br>Physical health
	Panel A: Males			
RDD wife earns more	-0.061*	0.004	0.009	-0.050***
	(0.032)	(0.007)	(0.029)	(0.014)
Observations	78,585	68,359	24,465	24,465
Mean of dep. var.	6.801	0.458	0	0
Std. dev. of dep. var.	2.152	0.498	1	1
	Panel B: Females			
RDD wife earns more	-0.083***	0.010	-0.050**	0.005
	(0.019)	(0.009)	(0.021)	(0.022)
Observations	78,608	68,370	24,412	24,412
Mean of dep. var.	6.827	0.459	0	0
Std. dev. of dep. var.	2.151	0.498	1	1

Notes - Standard errors are reported in parentheses and are clustered by relative income bins (in €1,000 intervals). All specifications include individual fixed effects. For each outcome, we used the optimal bandwidth using the mean square error (MSE) selection criteria and robust inference following Calonico et al. (2014).
*Significant at 10 per cent; ** Significant at 5 per cent; ***Significant at 1 per cent.

Table A.6: RD Estimates: Gender Norms and Well-being - Including Individual FE and Covariates

Satisfaction with	(1) Life	(2) Family	(3) Housework	(4) Work	(5) Sleep	(6) Childcare
Panel A: Males						
RDD wife earns more	-0.060**	-0.062**	-0.022	-0.065***	-0.069*	0.032
	(0.028)	(0.029)	(0.016)	(0.018)	(0.037)	(0.086)
Observations	78,735	40,224	48,831	74,540	35,891	20,074
Mean of dep. var.	7.187	8.279	6.868	7.108	6.902	7.075
Std. dev. of dep. var.	1.725	1.577	2.008	2.041	2.132	2.287
Panel B: Females						
RDD wife earns more	-0.086***	-0.046	-0.053***	0.000	-0.051	0.161**
	(0.019)	(0.030)	(0.019)	(0.031)	(0.050)	(0.063)
Observations	78,765	40,267	68,785	70,400	35,911	19,424
Mean of dep. var.	7.264	8.204	6.757	7.104	6.690	7.178
Std. dev. of dep. var.	1.732	1.655	1.906	2.061	2.214	2.408

Notes - Standard errors are reported in parentheses and are clustered by relative income bins (in €1,000 intervals). All models control for age and age squared, indicators for education for both partners, wage for both partners, number of children, as well as year, state and individual fixed effects. For each outcome, we used the optimal bandwidth using the mean square error (MSE) selection criteria and robust inference following Calonico et al. (2014).
*Significant at 10 per cent; ** Significant at 5 per cent; ***Significant at 1 per cent.

Table A.7: RD Estimates: Gender Norms and Health - Including Individual FE and Covariates

	(1)	(2)	(3)	(4)
Dep. var.:	Satisfaction with health	Poor health	Mental health	Physical health
	Panel A: Males			
RDD wife earns more	-0.049*	0.003	0.010	-0.046***
	(0.026)	(0.007)	(0.029)	(0.017)
Observations	78,585	68,359	24,465	24,465
Mean of dep. var.	6.801	0.458	0	0
Std. dev. of dep. var.	2.152	0.498	1	1
	Panel B: Females			
RDD wife earns more	-0.074***	0.009	-0.054***	0.002
	(0.024)	(0.010)	(0.021)	(0.022)
Observations	78,608	68,370	24,412	24,412
Mean of dep. var.	6.827	0.459	0	0
Std. dev. of dep. var.	2.151	0.498	1	1

Notes - Standard errors are reported in parentheses and are clustered by relative income bins (in €1,000 intervals). All models control for age and age squared, indicators for education for both partners, wage for both partners, number of children, as well as year, state and individual fixed effects. For each outcome, we used the optimal bandwidth using the mean square error (MSE) selection criteria and robust inference following Calonico et al. (2014).
*Significant at 10 per cent; ** Significant at 5 per cent; ***Significant at 1 per cent.
